# Limits on Replenishment of the Resting CD4^+^ T Cell Reservoir for HIV in Patients on HAART

**DOI:** 10.1371/journal.ppat.0030122

**Published:** 2007-08-31

**Authors:** Ahmad R Sedaghat, Janet D Siliciano, Timothy P Brennan, Claus O Wilke, Robert F Siliciano

**Affiliations:** 1 Department of Medicine, Johns Hopkins University School of Medicine, Baltimore, Maryland, United States of America; 2 Section of Integrative Biology, Center for Computational Biology and Bioinformatics, University of Texas at Austin, Austin, Texas, United States of America; 3 Institute for Cell and Molecular Biology, University of Texas at Austin, Austin, Texas, United States of America; 4 Howard Hughes Medical Institute, Baltimore, Maryland, United States of America; University of Pennsylvania School of Medicine, United States of America

## Abstract

Whereas cells productively infected with human immunodeficiency virus type 1 (HIV-1) decay rapidly in the setting of highly active antiretroviral therapy (HAART), latently infected resting CD4^+^ T cells decay very slowly, persisting for the lifetime of the patient and thus forming a stable reservoir for HIV-1. It has been suggested that the stability of the latent reservoir is due to low-level viral replication that continuously replenishes the reservoir despite HAART. Here, we offer the first quantitative study to our knowledge of inflow of newly infected cells into the latent reservoir due to viral replication in the setting of HAART. We make use of a previous observation that in some patients on HAART, the residual viremia is dominated by a predominant plasma clone (PPC) of HIV-1 not found in the latent reservoir. The unique sequence of the PPC serves as a functional label for new entries into the reservoir. We employ a simple mathematical model for the dynamics of the latent reservoir to constrain the inflow rate to between 0 and as few as 70 cells per day. The magnitude of the maximum daily inflow rate is small compared to the size of the latent reservoir, and therefore any inflow that occurs in patients on HAART is unlikely to significantly influence the decay rate of the reservoir. These results suggest that the stability of the latent reservoir is unlikely to arise from ongoing replication during HAART. Thus, intensification of standard HAART regimens should have minimal effects on the decay of the latent reservoir.

## Author Summary

Latently infected resting CD4^+^ T cells represent a stable reservoir for human immunodeficiency virus (HIV). When HIV-infected individuals are treated with highly active antiretroviral therapy (HAART), this latent reservoir decays slowly, with a half-life of up to 44 months. As a result, latently infected resting CD4^+^ T cells represent the major known barrier to eradication of HIV infection. Two factors are believed to contribute to the stability of the latent reservoir in the setting of HAART: replenishment by low-level viral replication and the intrinsic stability of resting memory CD4^+^ T cells. Unfortunately, it has not been possible to measure replenishment of this latent reservoir. In this study, we take advantage of a cohort of patients on HAART whose plasma virus consists largely of one (patient-specific) predominant plasma clone (PPC) that is grossly underrepresented in resting CD4^+^ T cells. We use the PPC as a label for ongoing viral replication by observing the accumulation of the PPC in resting CD4^+^ T cells over time in each patient. Analysis of the rate at which the PPC accumulates in resting CD4^+^ T cells allows us to quantitatively infer the maximum inflow of cells into the latent reservoir for HIV. Thus, we are able to provide the first quantitative constraint to our knowledge on the replenishment rate of the latent reservoir in the setting of HAART. Our results indicate that the rate of replenishment is very small and likely not a source of stability in the setting of HAART. These results have important implications regarding therapeutic options for purging the resting CD4^+^ T cell reservoir and curing HIV infection. Specifically, these results suggest that the intrinsic stability of latently infected resting CD4^+^ T cells, and not low-level viral replication, must be targeted therapeutically in order to achieve eradication of the latent reservoir.

## Introduction

The discovery of a stable latent reservoir for human immunodeficiency virus type 1 (HIV-1) [1–5] in resting CD4^+^ T cells uncovered a major obstacle to curing HIV-1 infection and revealed limitations of previous analytical predictions concerning eradication [6]. This reservoir persists despite years of highly active antiretroviral therapy (HAART) [7–9]. The observation that suspension of treatment leads to rapid rebound in viral load [10] may reflect the persistence of latently infected CD4^+^ T cells and possibly other viral reservoirs, as well as some degree of active viral replication that continues despite HAART [11–17].

The mechanism underlying the stability of the latent reservoir remains unclear. Some investigators have argued that residual viral replication continuously reseeds the latent reservoir [11,18,19], thereby providing long-term stability. With extremely sensitive methods, a low level of free virus can be detected in the plasma of patients on HAART who have suppression of viremia to below the limit of detection of ultrasensitive clinical assays [12,20–24]. In addition, many patients on HAART have transient episodes of detectable viremia termed blips [25–27]. These findings suggest that patients on HAART have a low level of viremia that may replenish the latent reservoir in resting CD4^+^ T cells through de novo infection of cells that then enter the reservoir. The other major explanation for the persistence of the latent reservoir is that the stability arises from the intrinsic dynamic properties of the latently infected cells. Because the reservoir consists of resting memory T cells [28], which form the basis of life-long immunity to previously experienced pathogens, the cells that harbor latent HIV-1 are fundamentally long-lived. Latently infected cells are protected from host immune responses because there is little or no transcription of viral genes in these cells [29,30]. The fact that some patients do not develop drug resistance despite long periods of HAART supports the idea that the virus can persist through mechanisms that do not involve continuous cycles of replication. Thus, the intrinsic stability of latently infected cells provides a plausible alternative explanation for the stability of the reservoir [21,31].

Curing HIV-1 infection will require elimination of the latent reservoir. It is therefore critical to understand which of these potential mechanisms are responsible for its stability. As a step in this direction, we have used mathematical modeling to understand the dynamics of the reservoir. Mathematical models have proven useful for analysis of several aspects of HIV-1 infection, including measurement of the turnover of different T cell subsets [32–35] and the response to therapy [6,36–44]. In this study, we take advantage of (and experimentally extend) a data set consisting of HIV-1 sequences from patients on HAART who maintain a distinctive pool of plasma virus [45]. In these patients, most of the residual viremia is comprised of a single predominant plasma clone (PPC) that is specific in sequence to each patient. Using this PPC as a label and a simple mathematical model, we take a maximum likelihood approach to quantitatively constrain the rate at which de novo infection replenishes the latent reservoir.

## Materials and Methods

### Experimental Procedures

The procedures for obtaining and analyzing the sequences used in this study have been described in detail elsewhere [45]. Briefly, we studied asymptomatic HIV-1-infected adults who had achieved suppression of viremia to <50 copies/ml on a stable HAART regimen for ≥6 mo and were willing to make frequent study visits. We previously described five patients who each had a PPC. For this present study, we exclude two of these patients (pt. 113 and pt. 139) due to a lack of sufficient follow-up sequence information beyond the intensive sampling period. The characteristics and treatment histories of the patients included in our analysis (pts. 135, 148, and 154) are representative of many HAART patients (exhibiting frequent and infrequent blips, on different HAART regimens with viral suppression from roughly 1.5 y to over 6 y) as previously described [46]. One of these patients, pt. 154, experienced (previous to this study) sequential failure of AZT monotherapy followed by failure of a three-drug HAART regimen and also exhibited multiple blips during the course of the study. Of all study participants, then, pt. 154 best represents the potential for ongoing viral replication (and therefore replenishment of the latent reservoir) in the setting of HAART.

To allow consistent amplification and sequencing of the small number of viral genomes present in the plasma of patients with viral loads below 50 copies/ml, plasma virus was first pelleted by ultracentrifugation, and then analyzed by limiting dilution reverse transcriptase (RT)-PCR, cloning, and sequencing using a previously described ultrasensitive genotyping method [45]. Viruses persisting in the resting CD4^+^ T cell reservoir were analyzed by a novel limiting dilution PCR assay [45]. Resting CD4^+^ T cells were purified from peripheral blood mononuclear cells by magnetic bead depletion as previously described [45]. As we have previously shown [3], these cells do not produce virus without stimulation and therefore by definition harbor latent virus. A segment of the *pol* gene encompassing all of protease and the first 219 amino acids of RT was amplified with nested PCR under limiting dilution conditions that ensure that each positive reaction has a ∼90% probability of being clonal. Products of positive PCR reactions were directly sequenced. Reactions containing more than one distinct template were identified by examination of chromatograms and excluded from the analysis.

As described previously, sequence analysis was carried out using techniques designed to avoid PCR resampling and PCR error [45]. Independent sequences that were identical to one another throughout this region of RT were identified using Varplot (kindly provided by Stuart Ray, Johns Hopkins University). Care was taken to avoid PCR errors in the sequence analysis. Proviral DNA samples were analyzed by limiting dilution PCR and direct sequencing. This approach has the advantage of eliminating PCR errors except for those that occur in the first or second cycle. For plasma virus, RT-PCR reactions were set up at limiting dilution, and positive reactions were cloned. Multiple clones were sequenced from each reaction. This allowed ready recognition of PCR errors as mutations appearing in only one clone from a set of clones obtained from a limiting dilution reaction. PCR errors were detected at a frequency that was no greater than the frequency expected based on a formal error analysis carried out on viral RNA from a cloned laboratory isolate of HIV-1 that was amplified under the same conditions [45]. These errors were reverted to patient consensus. Phylogenetic analysis was carried out on a segment of the RT coding region representing amino acids 38–219 as previously described [45]. For each time point, pie charts were constructed in which distinct taxa present at that time point were represented as separate slices, with the size of each slice being proportional to the number of independent clones isolated with that sequence.

The number of latently infected cells carrying replication-competent virus was quantified as previously described [47]. The number of HIV DNA-containing resting CD4^+^ T cells was determined by quantitative real-time PCR [45].

### Mathematical Models

To evaluate replenishment quantitatively, we used a simple mathematical model to represent the dynamics of the latent reservoir in patients on HAART who had suppression of viremia to <50 copies/ml and whose residual viremia was largely composed of a PPC. Because only a small number of plasma virus sequences can be obtained from a given blood sample when the viral load is below 50 copies/ml, patients underwent intensive (every other day) plasma sampling over a 3-mo period, and data from this period of intensive sampling were pooled. Consistent with our sequencing data, we assumed that there were no latently infected cells containing PPC at the beginning of the 3-mo sampling period. Otherwise, we did not make any assumptions about the origin of the PPC. We make the conservative assumption that the PPC first appears at the beginning of the observation period (time *t* = 0), despite the fact that the PPC could have been present before we detected it. Sequencing and fitness studies (including direct examination of *env, RT,* and *protease* genes) have revealed no significant functional differences between the PPC and other sequences from the relevant patient [45]. Furthermore, we have previously detected the PPC in resting CD4^+^ T cells [45] (Table 1), albeit at a very low frequency, suggesting that the PPC is replication competent. Therefore, we assume that the PPC is infectious and that once the PPC appears in the plasma, it should begin entering the latent reservoir if there is any inflow into the reservoir. We assume that the PPC has permanently disappeared from the plasma (and thus can no longer enter the reservoir) after a period of time, *t_e_*. In order to be maximally conservative, we allow *t_e_* to be at most the period of time that we experimentally observed the PPC in each patient's plasma (although it is not certain that the PPC has entirely disappeared at later time points). Because we use the PPC as a label for new entrants into the latent reservoir, we use a previously described mathematical model used for tracking labeled cells in studies of T cell dynamics in the setting of HIV infection [48,49]. Our model consists of two state variables representing the fraction of latently infected cells containing PPC proviruses (*L_1_*) and latently infected cells containing all other proviruses (*L_2_*) where

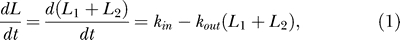
where *k_in_* is the rate constant for the entry of free viruses into the latent pool and *k_out_* is the decay rate of latently infected cells. Equation 1 has solution

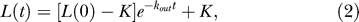
where we introduce 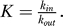


**Table 1:**
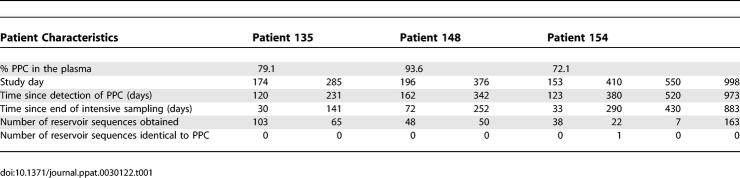
Presence of a PPC in the Plasma and Resting CD4^+^ T Cells of Patients on HAART

We extend this simple model of reservoir dynamics so that we consider *L*_1_(*t*) and *L*_2_(*t*) separately. We assume that these two populations of latently infected cells have the same kinetic properties and that the rate of replenishment of the latent reservoir is proportional to the fraction of the plasma virus of a given type. This model is described by a system of two ordinary differential equations,

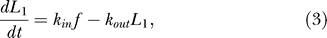


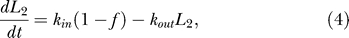
where *f* is the fraction of plasma virus that consists of the PPC. Equations 3 and 4 may be solved explicitly:

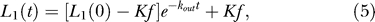


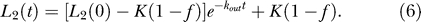



Using Equations 5 and 6, we can predict the dynamics of each latent reservoir population and therefore the fraction of latently infected cells in each pool at various time points while the PPC was present. At time points *t* after the PPC had disappeared from the plasma (at time *t_e_*), the two latent reservoir pools are described by the equations

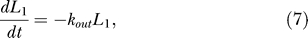


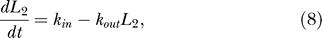
which have explicit solutions:

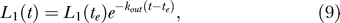


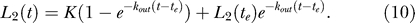



### Maximum Likelihood Estimation

The fraction of latently infected cells at time *t* that should contain the predominant plasma clone, *Φ_PPC_*, can be calculated from Equations 9 and 10 as

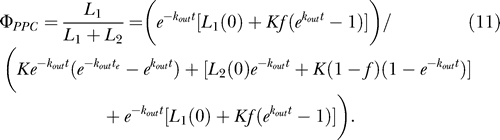



To be conservative, the initial number of latently infected cells containing PPC DNA (*L*_1_(0)) was set to zero for each patient. Because blood samples from time *t* = 0 were unavailable, the initial number of latently infected cells containing all other viral DNA sequences (*L*_2_(0)) was determined by extrapolation from experimental measurements by limiting dilution PCR at various time points for each patient (Table 2). We assume that the probability of finding *k* PPC cellular sequences out of *n* total latent reservoir sequences follows a binomial distribution, with the probability of success at time *t* set to the solution of Equation 11 at time *t*:

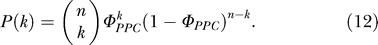



**Table 2:**
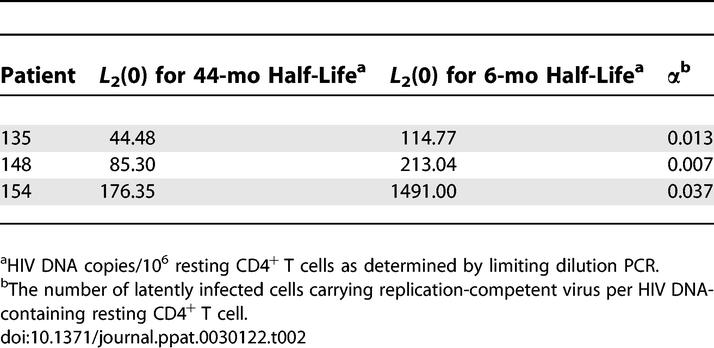
Initial Latent Reservoir Size, *L*_2_(0), under Different Reservoir Half-Life Assumptions for Patients on HAART

We used a maximum likelihood approach in order to find the value of *k_in_* most consistent with data collected from each patient [50]. Briefly, for specific values of *k_out_*, likelihood estimates, *P*(*k*), were calculated for a large range of *k_in_* values from Equations 12 using data collected from patients 135, 148, and 154. The *k_in_* value corresponding to the maximum likelihood estimate was designated as the most likely *k_in_* value.

We report analyses of patients that yielded informative results for *k_in_* (i.e., *k_in_* ≤ *k_out_L*).

All simulations and calculations were performed with MATLAB version 7.2.0.232 (http://www.mathworks.com/).

## Results

### Application of the Model towards Understanding Reservoir Dynamics

**Figure 1:**
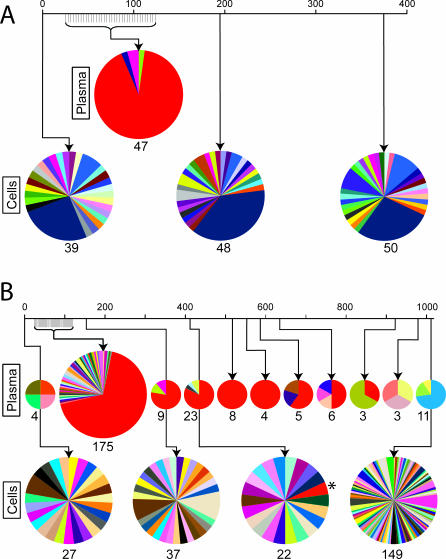
PPCs Show Limited Entry into Resting CD4^+^ T Cells of Patients on HAART (A and B) Results are shown for two patients, pt. 148 (A) and pt. 154 (B). The heterogeneity of the latent reservoir in comparison to the homogeneity of the plasma virus in these patients is represented with pie charts in which distinct genotypes are indicated in different colors. The PPC for each patient is shown in red. Intensive sampling of the plasma virus was carried out by sampling three times per week over a 3- to 4-mo period as indicated by the thin vertical marks on the time line. For pt. 154, additional samples of plasma virus (small circles) were obtained before and on several occasions after the period of intensive sampling. These document the persistence of the PPC for a minimum of approximately 900 d. Sampling of proviruses in resting CD4^+^ T cells was carried out before and on multiple occasions after the period of intensive plasma sampling. With one exception (*), all of the cellular sequences remained distinct from the PPC. The numbers below each circle represent the number of independent sequences analyzed. For pt. 154, the plasma samples after study day 900 were analyzed by RT-PCR of the *env* gene. Previously, linkage studies [45] allowed us to identify the PPC in the *env* sequences. Phylogenetic analysis of these sequences is described in detail elsewhere [45].

In a previous report, we described a population of HIV-1-infected individuals on HAART who had suppression of viremia to less than 50 copies/ml for an average of 34 mo [46]. Sequences of the residual plasma virus were obtained from these individuals by intensive sampling (three times per week) over a 3-mo period as well as at various intervals afterward for more than 1 y. Sequences from proviruses in resting CD4^+^ T cells (from our previous work, we know that these sequences are a reasonable surrogate for rescuable virus in the same population of cells [45]) were obtained at the beginning and end of the period of intensive sampling. In half of these patients, a single, homogenous but distinct viral sequence constituted a large fraction of the residual viremia but was profoundly underrepresented within sequences from resting CD4^+^ T cells at baseline (Figure 1). Linkage analysis suggested that this predominant plasma sequence actually represented a single PPC [45]. Because the PPC sequence was easily and specifically distinguishable from other sequences as a single sequence that was repeatedly detected in the plasma, this situation provided the ideal setting for determining whether the residual viremia could replenish the latent reservoir. Since most of the residual viremia was comprised of a unique genotype rarely found in resting CD4^+^ T cells, entry of a substantial number of these plasma viruses into the latent reservoir at later time points could be readily detected. Therefore, we were able to employ this unique plasma virus population as a functional label for measuring the rate of replenishment for the latent reservoir in the setting of HAART.

**Figure 2:**
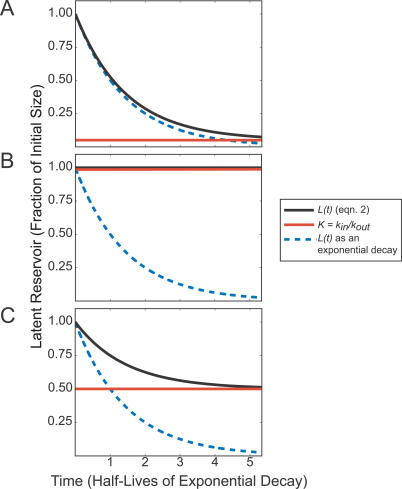
Dynamics of the Latent Reservoir (Black) *L* for the Three Described Regimes with Respect to the Steady State of *L* (Red) and a Purely Exponential Decay Defined by 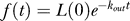
(Blue) (A) Regime 1; *k_out_L*(*t*) ≫ *k_in_*. (B) Regime 2; *k_out_L*(*t*) = *k_in_*. (C) Regime 3; *k_out_L*(*t*) ≈ *k_in_* and *k_out_L*(*t*) > *k_in_*.

In order to find the *k_in_* value most consistent with each patient's data, we take a maximum likelihood approach. To calculate a likelihood estimate for each *k_in_* tested, however, we must approximate *k_out_*. The dynamics of the latent reservoir may behave according to one of three regimes (described in Text S1), depending on the magnitude of *k_out_L*(*t*) compared to *k_in_* (Figure 2). In order to cover the most likely *k_in_* values for all possible levels of replication in the setting of HAART, we approximate *k_out_* for when the latent reservoir decays exponentially (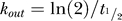 , where 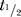 is an experimentally determined half-life of the latent reservoir) and for when the reservoir does not decay (*k_out_* = *k_in_/L*(0)) (regimes 1 and 2, respectively).

### Calculation of the Maximum Replenishment Rate of the Latent Reservoir

For each time point when cellular sequences were obtained, the most likely *k_in_* was chosen as the one with the maximum likelihood estimate, given the data (Table 1). We also calculated a 95% confidence interval around this value and use the upper bound of the 95% confidence interval as a conservative constraint on the maximum value of *k_in_*. Experimentally, at all but one time point, we found no PPC sequences in the latent reservoir. Consequently, the most likely value of *k_in_* calculated by the maximum likelihood approach was equal to zero cells per day. However, the fact that we found no PPC sequences in the latent reservoirs of our patients at these time points does not definitively indicate that there were no PPC sequences in the reservoir. In order to assign a more conservative frequency for the PPC sequence within the latent reservoir, we repeated all likelihood analyses with an add-one estimator [51], where 1 was added to the number of cellular PPC sequences obtained at each time point and 2 was added to the total number of cellular sequences obtained at each time point. Because the add-one approach already overestimates the presence of the PPC in the latent reservoir, the most likely *k_in_* calculated with the add-one estimator (mean value and not upper bound of the 95% confidence interval) is used as a conservative constraint on the reservoir inflow rate. To calculate the flow of replication-competent viruses into the latent reservoir, each patient's calculated *k_in_* values were multiplied by *α,* the experimentally determined ratio of cells carrying replication-competent virus to cells carrying HIV-1 DNA (Table 2). From this point forward, *k_in_* will refer to the flow of replication-competent virus into the latent reservoir.

For the case when *k_out_* dominates reservoir decay, we set *k_out_* = 0.000525 day^−1^, which reflects the previously reported 44-mo half-life of the latent reservoir. Initially, we assumed that the PPC was present for only the 90-d intensive sampling period despite the fact that two of the three patients maintained the PPC beyond this time. Under these assumptions, our maximum likelihood analysis found a most likely reservoir inflow rate of 0 cells/day, with an upper 95% confidence bound of 151 cells per day in pt. 135 (Table 3). The add-one estimator predicted a most likely *k_in_* of 94 cells per day in this patient. Similar rates were observed for pt. 148, and slightly higher rates were observed for pt. 154. Pts. 135 and 154 both maintained the PPC in the plasma at levels comparable to or above *f* beyond the intensive sampling period (until study day 174 for pt. 135 and day 922 for pt. 154) (unpublished data). To more accurately reflect the persistence of the PPC, we repeated the maximum likelihood analysis and assumed the PPC was present from time zero until the last study day when the PPC was observed in the plasma. Under this assumption, we again found a most likely inflow rate of 0 cells/day for pt. 135. Using the upper bound of the 95% confidence interval, we found that the data are consistent with *k_in_* up to 111 cells per day (Table 3). The add-one estimate predicts the data to be consistent with *k_in_* up to 70 cells per day in this patient. Similar or slightly higher rates were calculated for the other patients.

**Table 3:**
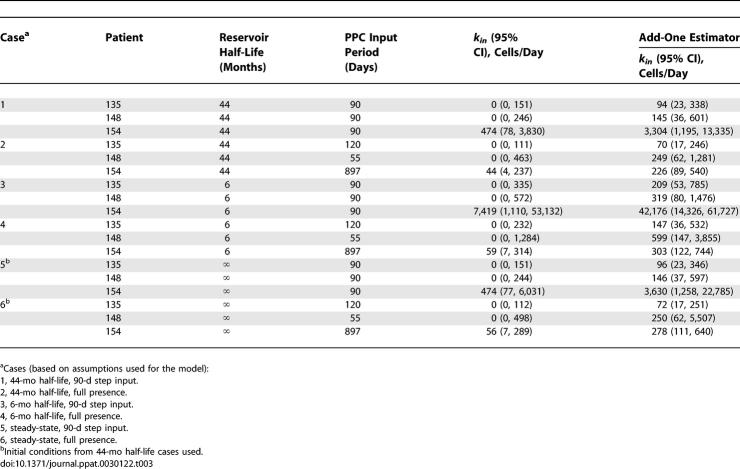
Estimation of Patient-Specific, Maximum Reservoir Inflow Rates

Because the true decay rate of the latent reservoir remains controversial, we repeated the above analyses for *k_out_* = 0.0039 day^−1^ (Table 3), which reflects a 6-mo half-life for the latent reservoir, the fastest reservoir decay rate reported [19,52]. By performing our analysis for the slowest (44-mo half-life) and fastest (6-mo half-life) reported reservoir decay rates, we cover the entire range of possible *k_in_* values. With a 6-mo reservoir half-life and the assumption of the 90-d step input of the PPC, *k_in_* was constrained to up to 335 cells per day by the data for pt. 135, with the add-one estimate constraining *k_in_* to 209 cells per day. Repeating the analysis for experimentally determined patient-specific PPC inputs, we found *k_in_* to be constrained to up to 232 cells per day by the data and 147 cells per day with the add-one likelihood estimate for this patient.

In our analysis above, we used an approximation of *k_out_* based on an assumed exponential decay of the latent reservoir (regime 1). The true range of *k_in_* values for each patient is bounded by the range of *k_in_* values calculated for regime 1 (latent reservoir decays exponentially) and regime 2 (latent reservoir is at steady state). We therefore calculated *k_out_* for the latent reservoir at steady state and repeated the analyses described above (Table 3). We initially assumed a 90-d step input of the PPC into the plasma of each patient and found that our analysis found a most likely reservoir inflow rate of 0 cells/day, with an upper 95% confidence bound of 151 cells per day. The add-one estimator predicted that the data are consistent with a most likely *k_in_* equal to 96 cells per day. When we extended the period of PPC presence in the plasma until the last study day when it was last observed for each patient, we found that the data constrained *k_in_* to be up to 112 cells per day for pt. 135, with the add-one estimator constraining *k_in_* to be up to 72. The *k_in_* estimates for the steady-state assumption are not significantly different from the *k_in_* estimates made above with *k_out_* dictating the reservoir decay rate, suggesting that the calculated maximum *k_in_* values consistent with all of the data are good approximations for the true maximum rate of inflow into the latent reservoir.

### The Maximum Daily Flow of Cells into the Latent Reservoir during HAART Is a Small Fraction of the Patients' Reservoir Size

It is also helpful to view the maximum absolute flow rate into the reservoir in the context of the overall reservoir size in each patient. We approximate the percentage of the total reservoir that daily reservoir inflow represents by normalizing the daily reservoir inflow (the upper bound of the 95% confidence intervals for each *k_in_* calculated based on the pure data or the most likely *k_in_* calculated with an add-one estimate in Table 3) with the starting number of replication-competent cells in the latent reservoir (*α*(*L*_1_(0) + *L*_2_(0))) (Table 4). Our calculations indicate that for each patient, maximum daily flow of infected cells into the reservoir is very small compared to the total reservoir size. In fact, most maximum *k_in_* calculations were on the order of 0.01%–0.1% of the starting total reservoir size and even as low as < 0.001%. Because the total reservoir size decreases over time and we assume a constant *k_in_*, the values in Table 4 increase over time. On the time scale of when samples were taken from patients, however, none of the values in Table 4 would increase by more than 2-fold if assuming a 44-mo reservoir half-life, and most would not increase by more than 4-fold if assuming a 6-mo reservoir half-life.

**Table 4:**
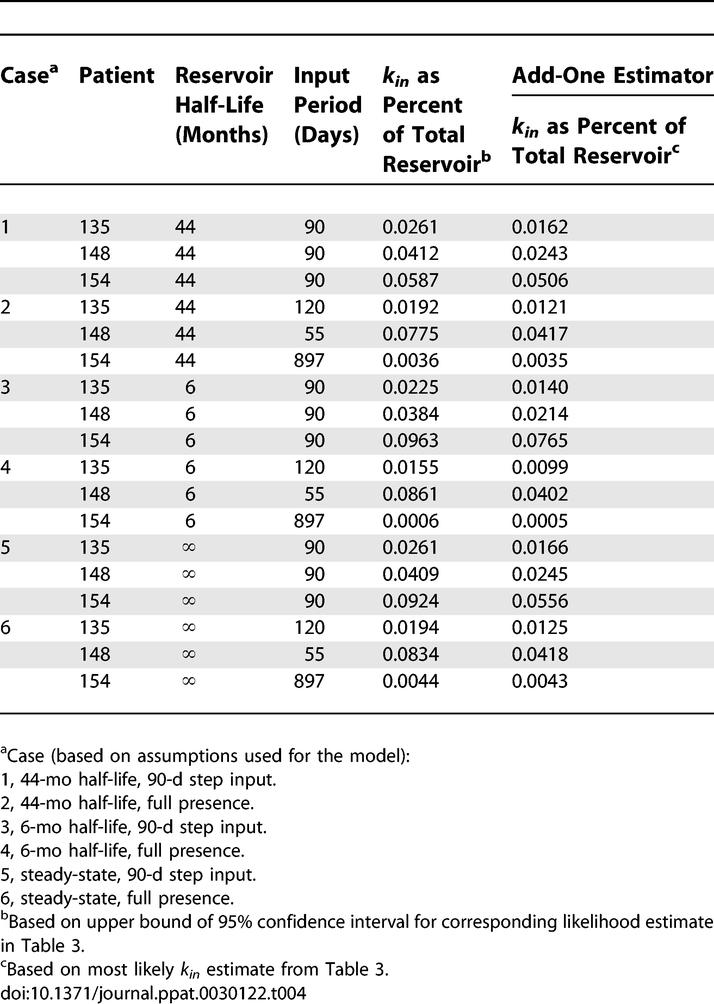
Patient-Specific Maximum Daily Reservoir Inflow as a Percent of the Total Latent Reservoir Size

### The Maximum Daily Flow of Cells into the Latent Reservoir during HAART Is Considerably Reduced Compared to the Pre-HAART Reservoir Inflow

We also consider whether our predicted maximum daily inflow of cells into the latent reservoir during HAART reflects a reduction from the predicted pre-HAART inflow. We have previously described how long each patient in this study had consistently suppressed viremia (79 mo for pt. 135, 35 mo for pt. 148, and 17 mo for pt. 154 from Table 1 of [45]) prior to enrollment in our study, and we have measured the size of each patient's latent reservoir at the beginning of our study (Table 2). From these values, and assuming a reservoir half-life of 44 mo (corresponding to *k_out_* = 0.000525 day^−1^), we are able to back calculate the most recent size of each patient's pre-therapy latent reservoir, 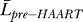 . The steady-state, pre-HAART reservoir inflow for each patient may be calculated by 
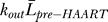
 . Based on this calculation and our lowest predicted upper bound on *k_in_* for each patient in Table 3, we find that HAART has reduced the daily inflow into the latent reservoir by *at least* (since we calculate the *maximum* reservoir inflow rate) a factor of 10.7 for pt. 135, 2.2 for pt. 148, and 18.8 for pt. 154, if we assume a 44-mo half-life for the resting CD4^+^ T cell reservoir. These fold reductions are several orders of magnitude larger if we assume a 6-mo half-life. These results indicate that HAART can drastically reduce the flow of cells into the resting CD4^+^ T cell reservoir.

## Discussion

The latent reservoir for HIV-1 in resting CD4^+^ T cells is the primary known barrier to eradication of HIV-1 infection. Therefore, eradication of HIV-1 infection depends on successful purging of the latent reservoir from an infected individual. Unfortunately, experimental evidence has shown that the latent reservoir is highly stable. Whereas the half-lives of other types of infected cells in the setting of HAART are on the order of days to weeks, the half-life of the latent reservoir has been reported to be on the order of months to years [7–9]. The longevity of the reservoir requires HIV-1-positive individuals to remain on HAART for their entire lives [53].

The basis for the stability of the latent reservoir is controversial. Some reports have shown an increase in the reservoir decay rate with an intensified HAART regimen [18], suggesting that low-level viral replication continuously replenishes the reservoir [11,18,19]. Because the latent reservoir resides within memory CD4^+^ T cells, which are inherently long-lived cells, some have hypothesized that the latent reservoir's longevity stems from its intrinsic stability [7–9,20,28]. Longitudinal studies of patients on standard HAART have shown no generation of new drug-resistant virus in plasma, suggesting a halt in viral replication [21]. These studies support the notion that the latent reservoir is intrinsically stable, consistent with the known properties of memory CD4^+^ T cells. It is difficult to find direct, experimental support for either argument, however, due to the lack of a readily accessible experimental model.

Opportunities do arise when patient-derived data may be used to gain unique insights into the latent reservoir. In this study, we offer a quantitative glimpse into the replenishment of the latent reservoir in the setting of HAART. Although several studies have suggested that reservoir replenishment might occur during HAART [11,18,19], there has been no quantitation of the replenishment rate. This has been due in part to the fact that there is no way of uniquely labeling latently infected resting CD4^+^ T cells and subsequently following that label. In this study we take advantage of a previously reported phenomenon where a unique, patient-specific viral sequence (PPC) dominated the residual plasma virus but could not be readily found in the patient's activated or resting CD4^+^ T cells [17,45]. For our study, we use and extend a previously reported, exhaustive data set of plasma and proviral sequences [45]. We hypothesized that replenishment of the latent reservoir by viral replication in the presence of the PPC would eventually lead to incorporation of the PPC into the latent reservoir. We used a simple mathematical model of latent reservoir dynamics to constrain the maximum rate of reservoir replenishment by viral replication in the setting of HAART.

Our model was constructed with as few assumptions as possible regarding the nature of reservoir dynamics. Our model does rely on the assumption that the PPC is replication competent, or at least capable of infecting and integrating into the genome of a CD4^+^ T cell. Our previous study strongly suggested that each patient's PPC was not different than other patient-specific plasma virus sequences in functionality (by direct examination of *env, RT,* and *protease* genes) or resistance to antiretroviral drugs or host-mediated immune responses [45]. Furthermore, detection of the PPC at a low frequency in resting CD4^+^ T cells [45] was highly suggestive of the idea that the PPC is replication competent. We therefore considered the PPC to be the same as other viral sequences with respect to replenishing the reservoir. However, even a non-infectious PPC would be indicative of minimal viral replication in the setting of HAART, because the PPC comprises such a large portion of the plasma virus (and it would be unlikely that all other plasma virus is produced from viral replication with no release from the reservoir). All other assumptions that were made would only artificially increase the calculated maximum reservoir replenishment rate. In particular, we assume that the PPC is first present in the plasma when we first detect it experimentally, whereas it may easily have been present in the patient much earlier. We have also assumed that any PPC sequence found in resting CD4^+^ T cells is due to infection of that cell after initiation of HAART. Clearly this is not the only possibility, as a PPC sequence may have entered a resting CD4^+^ T cell before HAART was initiated.

By applying this approach to data derived from three independent HIV-1-infected individuals on HAART who exhibited a PPC, we have been able to quite conservatively constrain the replenishment rate of the latent reservoir to be at most on the order of 100 cells carrying replication-competent virus per day. Given that the average size of the latent reservoir is approximately one million cells [1], we have therefore constrained the daily reservoir inflow to be approximately 0.01%–0.1% of the total reservoir size for the average HIV^+^ patient on HAART. Our results predict a substantial reduction in the reservoir inflow in the setting of HAART compared to pre-HAART levels. While we could not demonstrate a drastic reduction in reservoir inflow for pt. 148 due to the limited number of available sequences, we were able to show in other patients that HAART reduces the reservoir inflow by at least 10- to 20-fold from pre-HAART levels. Given pt. 154′s treatment history of frequent blips suggestive of low-level viral replication, that we are able to predict a ∼20-fold reduction in reservoir replenishment suggests that HAART would reduce the reservoir replenishment rate by even more in patients like pt. 135 and pt. 148, who exhibit no signs of potential viral replication. Subsequent analyses and procurement of additional sequence data may allow us to reduce the maximum replenishment rate of the reservoir even further. Our present results, however, do not establish whether or not there is any replenishment of the latent reservoir by low-level viral replication in the setting of HAART. Our analysis uses patient-derived data to conservatively constrain the *maximum* replenishment rate of the latent reservoir in the setting of HAART.

Of the three patients in our study, we detected the PPC in the resting CD4^+^ T cell compartment of one. We believe that the infrequent detection of PPC in resting CD4^+^ T cells of our study participants reflects the following: 1) few resting CD4^+^ T cells contain PPCs, and 2) replenishment of the latent reservoir in the setting of HAART must be slow, which is consistent with our results. Furthermore, while we can only infer a maximum daily inflow into the latent reservoir (since we cannot sequence the entire latent reservoir, but rather only a sample of the latent reservoir at each time point), the actual replenishment rate may easily be much lower than our calculated maximum replenishment rate and may even be zero. Finally, because the data have constrained the maximum reservoir inflow rate to be small compared to the total reservoir size, it may be that the flow of new cells into the reservoir does not significantly affect the decay rate of the latent reservoir in these patients (regime 1 versus regimes 2 and 3 described above).

The finding that the daily inflow into the reservoir is small compared to the overall reservoir size suggests that the decay of the reservoir in our patients (who have all been on HAART for several years) is more likely determined by *k_out_* (the intrinsic decay rate of latently infected cells) and not *k_in_* (new entry into the reservoir). Our model, however, predicts that the latent reservoir will eventually achieve a new steady-state level of *k_in_*/*k_out_*. If there is an inflow into the latent reservoir, despite HAART, further intensification of HAART may reduce the steady-state latent reservoir level or even lead to eradication of the reservoir. Because our results indicate that inflow into the reservoir must be very small and therefore probably does not affect the reservoir decay rate, an immediate benefit from HAART intensification may not be apparent. In fact, our results suggest that HAART intensification could *at best* cause the latent reservoir to decay with a half-life of on average 43.5 to 118 mo (Text S2).

The results of our analysis are important on a practical level. It has been suggested that intensification of HAART may stop residual viral replication in the setting of standard HAART and increase the decay rate of the latent reservoir [18]. HAART intensification poses a problem for physicians and patients because intensified HAART also leads to intensified drug toxicities such as lipodystrophy, hepatotoxicity, and gastrointestinal symptoms [54–56]. Drug toxicity not only leads to patient morbidity but also motivates non-adherence with subsequent development of drug resistance by the virus. Further analyses may enlighten the cost versus potential benefit of intensified HAART and hopefully maximize the clinical benefit or treatment for patients with minimal morbidity.

## Supporting Information

Text S1 Appendix I (56 KB DOC)

Text S2 Appendix II (21 KB DOC)

## Abbreviations

HAART: highly active antiretroviral therapy
HIV: human immunodeficiency virus
PPC: predominant plasma clone
RT: reverse transcriptase
