# Supplementary material for: Limits on Replenishment of the Resting CD4+ T Cell Reservoir for HIV in Patients on HAART
Source: PLoS Pathog. 2007 Aug 31;3(8):e122. doi: 10.1371/journal.ppat.0030122 (PMC1959378; doi:10.1371/journal.ppat.0030122)
Supplement: Text S1 — (56 KB DOC) [file ppat.0030122.sd001.doc]

**Appendix I**

In our simple mathematical model, the dynamics of the total reservoir ($L$) are controlled by the choices for $k_{in}$, $k_{out}$ and $L(0)$, the total reservoir size at the beginning of the observation period (*t*=0). As time goes on (for large *t*), $L(t)$ approaches its steady-state level of *K*. If $L(0)$ is larger than *K*, then $L(t)$ will decay towards *K*. Likewise, if $L(0)$ is smaller than *K*, $L(t)$ will grow towards *K*. Because we are modeling the dynamics of the latent reservoir in the setting of HAART and there is strong evidence that the reservoir does not grow in this setting [7–9], we will not consider the latter case of $L(0)<K$. In this context, the behavior of $L(t)$ falls within one of three regimes, depending on the choices of $k_{in}$and $k_{out}$ (Figure 2):

1. When $k_{out}L(t)>>k_{in}$, $L(t)$ decays in an approximately exponential manner with a half-life of $\ln(2)/k_{out}$.
2. When $k_{out}L(t)=k_{in}$, $L(t)$ is at its steady-state level and will not change (half-life of the reservoir equals infinity). Because $L(0)=K$, the system starts at steady-state and remains there so a half-life for the decay of$L(t)$ cannot be defined in this regime.
3. When $k_{out}L(t)\approx k_{in}$ and $k_{out}L(t)>k_{in}$, $L(t)$ decays towards *K* but cannot be approximated well as an exponential decay so a half-life cannot be defined in this regime.

In order to perform a maximum likelihood analysis for determination of $k_{in}$, we must approximate $k_{out}$. The most likely $k_{in}$ for a specific $k_{out}$under regime (3) will fall between the most likely $k_{in}$ calculated under regime (1) for the same $k_{out}$ and that calculated for regime (2). Therefore we cover most likely $k_{in}$ values for all possible levels of replication in the setting of HAART by finding the most likely $k_{in}$ values for $k_{out}$ values approximated by regimes (1) and (2).
