# Supplementary material for: Limits on Replenishment of the Resting CD4+ T Cell Reservoir for HIV in Patients on HAART
Source: PLoS Pathog. 2007 Aug 31;3(8):e122. doi: 10.1371/journal.ppat.0030122 (PMC1959378; doi:10.1371/journal.ppat.0030122)
Supplement: Text S2 — (21 KB DOC) [file ppat.0030122.sd002.doc]

**Appendix II**

We can also consider the other extreme where the effects of viral replication on the decay of the reservoir in the setting of HAART are maximized (i.e. the latent reservoir is at steady-state), described by regime (2). In this case the intrinsic decay rate of the reservoir is balanced by inflow into the reservoir. In this scenario intensification of HAART could maximize the reservoir decay rate by reducing $k_{in}$ to zero. We could therefore predict the maximum decay rate of the latent reservoir under intensified HAART as: $k_{out}$= $k_{in}^{calculated}$/$(L_{1}(0)+L_{2}(0))$ , where $k_{in}^{calculated}$ is the maximum calculated inflow rate. The patient-averaged $k_{out}$ , under steady-state cases 5 and 6 (Table 3) for both standard and add-one likelihood estimates, was found to be between 0.000195 and 0.000531 day^-1^. Thus in the worst case scenario where the reservoir remains at steady-state due to ongoing viral replication in the setting of standard HAART, our results suggest that HAART intensification could *at best* cause the latent reservoir to decay with half-life of on average 43.5 to 118 months.
